# Travelling Thoughts

**Published:** 1871-10

**Authors:** 


### TRAVELLING THOUGHTS.

1. Eat regularly thrice a day, and never between meals.

2. Take with you one third more money than you calculate on spending.

3. Take small bills, rather than large, to avoid having bad money passed on you, in change.

4. Aim to be at your place of starting at least ten minutes before the time, and grow merry and wise at the contemplation of the splutterings and mishaps of those who come in at the last minute, and half a minute later.

5. See that your baggage is on the conveyance before you are yourself.

6. Remember that you make your character as you go along, by the quiet courtesy of your manners.

7. Only boors are boisterous.

8. Do not let the servants excel you in patience and politeness.

9. "Please" should commence every request, and "Thanks" end every service done.

10. A lady is always gentle; a gentleman always composed.

11. Never argue on any subject, if there is more than one present besides yourself.

12. Never fail to set that person down as ignorant or low bred, who by word, or look, or gesture, disparages a woman, a clergyman, the Bible, or the Sabbath day.

